# Carbon (δ^13^C) and Nitrogen (δ^15^N) Stable Isotope Signatures in Bat Fur Indicate Swarming Sites Have Catchment Areas for Bats from Different Summering Areas

**DOI:** 10.1371/journal.pone.0125755

**Published:** 2015-04-29

**Authors:** Jordi L. Segers, Hugh G. Broders

**Affiliations:** Department of Biology, Saint Mary’s University, Halifax, Nova Scotia, Canada; Università degli Studi di Napoli Federico II, ITALY

## Abstract

Migratory patterns of bats are not well understood and traditional methods to study this, like capture-mark-recapture, may not provide enough detail unless there are many records. Stable isotope profiles of many animal species have been used to make inferences about migration. Each year *Myotis lucifugus* and *M*. *septentrionalis* migrate from summering roosts to swarming caves and mines in the fall, but the pattern of movement between them is not well understood. In this study, fur δ^13^C and δ^15^N values of 305 *M*. *lucifugus* and 200 *M*. *septentrionalis* were analyzed to make inferences about migration patterns between summering areas and swarming sites in Nova Scotia, Canada. We expected that there would be greater variability in δ^13^C and δ^15^N among individuals at swarming sites because it was believed that these sites are used by individuals originating from many summering areas. There was extensive overlap in the standard ellipse area, corrected for small sample sizes (SEA_c_), of bats at swarming sites and much less overlap in SEA_c_ among groups sampled at summering areas. Meaningful inference could not be made on *M*. *septentrionalis* because their low variation in SEAc may have been the result of sampling only 3 summering areas. However, for *M*. *lucifugus*, swarming sites had larger SEA_c_ than summering areas and predictive discriminant analysis assigned swarming bats to multiple summering areas, supporting the contention that swarming bats are mixed aggregations of bats from several summering areas. Together, these data support the contention that swarming sites have catchment areas for bats from multiple summering areas and it is likely that the catchment areas for swarming sites overlap. These data suggest that δ^13^C and δ^15^N profiling of bat fur offer some potential to make inferences about regional migration in bats.

## Introduction

Migration is a key life history strategy among animals to cope with seasonal variation in resource abundance [1,2]. Migratory animals depend on multiple ranges (e.g., wintering, summering and stop-over sites) and therefore these species may be more vulnerable to environmental perturbations than sedentary species [2–4]. For example, anthropogenic alteration within a species’ breeding range may cause decreased breeding success [5] and disturbance at stop-over sites may affect the ability to replenish fat reserves [6,7]. Hence, there is great interest in understanding the dynamics of migration [8,9].

Traditionally, direct methods of capture-mark-recapture or animal tracking are used to study migration [10,11], but these methods have many limitations when studying small volant animals. Recaptures over vast distances are sparse, radio telemetry often has limited broadcasting range [12], satellite tags are too heavy for some species [13] and geolocators rely on daylight for geo-referencing locations [14]. More recently, advances in DNA profiling technology and population genetic theory are being applied to indirectly study migration [15–18] as population level data may be used to characterize patterns of gene flow and estimate effective population size [17,18]. However, different markers often yield conflicting results [17] and often do not provide sufficient geographic resolution [19].

Another indirect means to study animal migration is the spatio-temporal characterization of stable isotopes. Stable isotopes are non-radioactive, naturally occurring forms of elements that vary in atomic weights. Stable isotope profiles of organisms are, in part, a function of the isotopic composition of the lower trophic level from which they derive their diet and, as such, the profiles change depending on seasonal fluctuations and abundance of prey species. Stable isotope profiling of an organism’s tissue may provide insight into its origin and trophic level (e.g., habitat type, climatic condition) [12,18,20]. Hence, stable isotopes may be used as markers representing, at least in part, the time and location of an organism’s origin [21]. In biology, stable isotopes are most often used to understand trophic dynamics and diet [22–24]. However, in the last decade some stable isotopes have been used to make inferences about migration patterns [25–28].

Typically, when using stable isotopes to study movement of animals, stable hydrogen (*δ* ^2^H) or sulphur (*δ* ^34^S) isotope ratios are used. Hydrogen isotopes can be useful for making inferences about long-distance migration because *δ* ^2^H in animal tissues reflect local precipitation patterns, which differ over latitudinal gradients and can therefore be used to trace origins of migrating species [29]. Also, *δ* ^2^H patterns differ seasonally and between marine and terrestrial sites [12]. Sulphur isotopes are used primarily to make inferences about marine and marsh food webs [30], but Zazzo et al. [31] demonstrated their use for terrestrial animals, finding a correlation between *δ* ^34^S and distance from the coast. Scale of movement of short-distance migratory species is likely too small to use *δ* ^2^H for identifying movement patterns. Sulphur isotopes may be effective, especially in coastal areas, but require a labour and cost intensive baseline study prior to analysis [31]. Most often, carbon (*δ* ^13^C) and nitrogen (*δ* ^15^N) stable isotope ratios are used in studies on terrestrial or freshwater food webs [20,22,23], but multivariate analysis has been applied to these to study movement of marine species [32–34] and birds [35]. In the environment *δ* ^13^C varies in plant tissue according to photosynthetic rate and decreases with increasing latitude, *δ* ^15^N varies in plant tissue according to how nitrogen is fixed and wet sites are more enriched in *δ* ^15^N than dry sites [12].

Though it is known that many bat species migrate [36], details of the dynamics for most species are poorly understood. Despite obstacles and limitation, some studies have successfully made inferences about bat migration ecology using mark-recapture techniques with a large number (3000–73000) of captured animals [37–39]. Bat migration can be classified, depending on species, as long-distance migration (> 500 km) and regional migration (100–500 km) [16,36,40]. Examples of long-distance migrants in North America are the hoary bat (*Lasiurus cinereus*) and red bat (*L*. *borealis*), which are believed to fly > 1000 km between their temperate summering area and their southern wintering site [36,41,42]. Straw-coloured fruit bats (*Eidolon helvum*) from Africa, large enough for satellite tracking studies, migrate over 2000 km [13]. The little brown bat (*Myotis lucifugus*) and the northern long-eared bat (*M*. *septentrionalis*) are regional migrants from North America that migrate from winter hibernacula to summering areas. Davis and Hitchcock [37] described movements of *M*. *lucifugus* up to 275 km from hibernacula to summer colonies and Norquay et al. [39] report recaptures of *M*. *lucifugus* as far as 569 km from their initial capture site. *Myotis septentrionalis’* regional movement patterns are less studied, but Nagorsen and Brigham [43] report records of *M*. *septentrionalis* traveling 56 km between summering areas and hibernacula.

From August to October *M*. *lucifugus* and *M*. *septentrionalis* migrate and congregate at the entrance of caves and mines (swarming) before going into hibernation. This swarming behaviour may serve multiple purposes including mating and other social behaviours [44]. After swarming they use natural caves and abandoned mines to hibernate and after leaving their hibernacula in spring they migrate to summering areas [45–49] to which they may have long-term fidelity [47,50].

Stable isotopes in keratinous tissues are arguably the best for studying seasonal movement patterns of animals [12]. Unfortunately, few studies on the moult of bats exist and for many species moult time is not known or data are sporadic and inconsistent. Jones and Genoways [51] describe one record of a male *M*. *lucifugus* moulting early July, but no others showed any signs of moulting during that study. Fraser et al. [25] suggested that tri-coloured bats (*Perimyotis subflavus*) moult between June and October. Other bat species have been observed to moult between July and mid-August [52–54]. Fraser et al. [55] suggest that fur from adult male bats taken dorsally most likely represents site of summer residency. Bats spend most of their foraging-time at summering areas, thus energy-intake occurs mostly here and intraspecific variation in isotopic signatures of bat fur should largely be a function of diet and environmental variation at the location of new fur growth, which presumably occurs at these summering areas. Following this concept, Baerwald et al. [56] used *δ* ^2^H, *δ* ^13^C and *δ* ^15^N in fur of long-distance migratory bats to make inferences about summering region origins.

Abundance of different stable isotopes in nature varies because of biological (terrestrial vs. aquatic) and anthropogenic (e.g., agriculture, pollution) factors. Through spatial variation in stable isotope profiles in the environment, inferences may be made about population-level movements [12,29]. Spatial variation in isotopic composition in the environment, and thus also in bat fur, may be smaller among local colonies. Stable isotopes of carbon and nitrogen are typically used for dietary studies and there can be both interspecific and intraspecific variation [12,20,29]. For example, because the diet of *M*. *lucifugus* is composed mainly of insects from aquatic systems their profiles may be different from *M*. *septentrionalis*, which mainly feeds on insects from a terrestrial origin [57], due to differences between stable isotope ratios in aquatic and terrestrial ecosystems [47,49]. Body size, especially in predatory animals, is often correlated with niche breadth where larger species are capable of eating both large and smaller prey and smaller species can only consume smaller prey items [58,59]. Not only is this the case interspecifically but also on an intraspecific level where larger or older individuals may have a greater niche width than smaller or younger individuals [60]. Similarly, intraspecific variation may occur because, through intersexual differences in foraging ecology, males and females may consume different prey from different site-types [45,46,48,49,61].

Several summering areas and swarming sites for *M*. *lucifugus* and *M*. *septentrionalis* have been identified in Nova Scotia, Canada [61,62], but little is known about where animals that summer in one place migrate to at the end of the season for swarming. The goal of this study was to test whether *δ* ^13^C and *δ* ^15^N in the fur of bats may be used to make inferences about their migration patterns. Specifically, we were interested in characterizing the spatial variability in the stable isotope patterns of *δ* ^13^C and *δ* ^15^N in bats at summering areas and swarming sites in Nova Scotia to make inferences about variation in the origin of bats at swarming sites. We also wanted to test whether any intraspecific variation in isotope signatures could be explained by sex or body size. We formulated three predictions regarding isotopic variation:

1. We predicted that colonies nearer to one another would be more similar in isotopic niche than those further apart.
2. Since *δ* ^13^C and *δ* ^15^N data are generally used to make inferences about diet, and *M*. *lucifugus* and *M*. *septentrionalis* are thought to occupy different dietary niches [45,46,48,49,57,61], it was predicted that there would be interspecific variation in isotopic signatures.
3. Finally, because swarming sites were predicted to have been used by bats from multiple summering areas, we predicted swarming sites to show more isotopic variation than summering areas and that the variation between summering areas is greater than between swarming sites.

## Materials and Methods

### Sample collection

**Fig 1:**
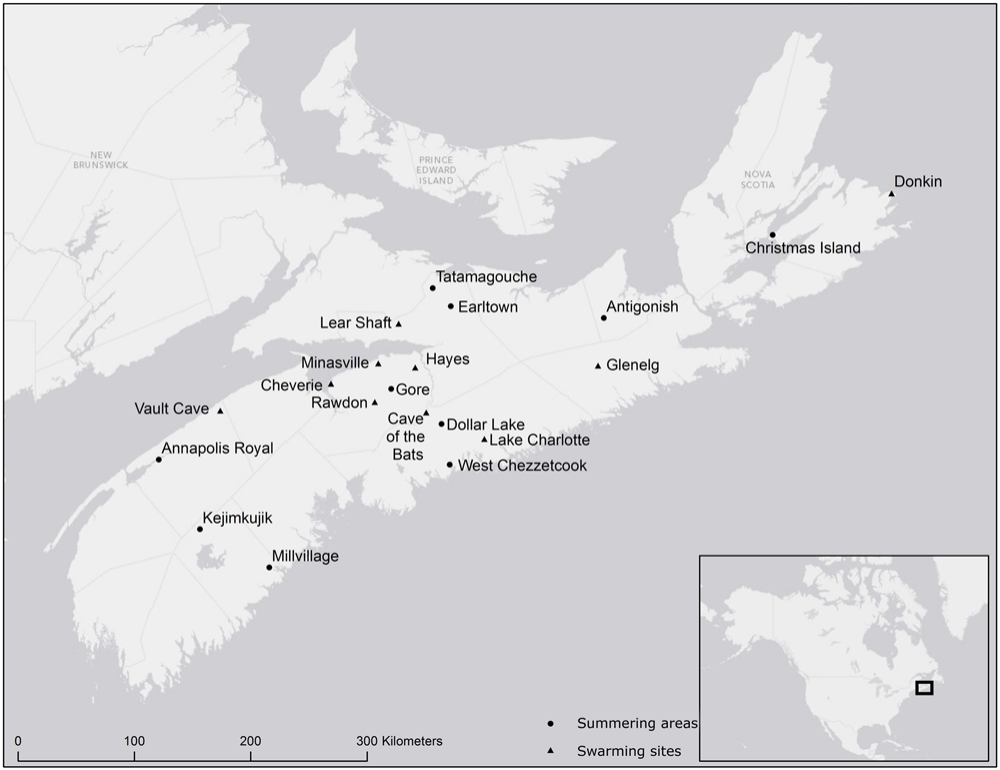
Sampling sites in Nova Scotia; circles indicate summering areas and triangles indicate swarming sites.

*Myotis lucifugus* and *M*. *septentrionalis* were captured from 2001 to 2013 using mist nets (Avinet Inc, Dryden, New York, USA) and harp traps (Austbat Research Equipment, Lower Plenty, Victoria, Australia). Bats were identified to species, sexed and aged, and forearm measurements were taken with calipers to the nearest 0.01mm. Fur samples were collected by cutting a small amount (≈1.4 mg) from between bats’ scapulae with cuticle scissors. Samples were stored in 1.5 ml eppendorf tubes and archived at -20°C. Summering bats were captured between May 19 and August 7 and swarming bats were captured between August 11 and October 3. Nets and traps were set one hour before sunset and left open for at least three hours. Methods for the capture and handling of bats were approved by the Saint Mary's Animal Care Committee and under permit from the Nova Scotia Department of Natural Resources. Samples for analysis were selected to represent a wide geographic area within Nova Scotia (Fig 1) with variability among environment types (e.g., terrestrial, aquatic, marine, agriculture, forests), and individuals were selected to represent the breadth of variation in forearm length at each site in the event there may be an effect of body size on stable isotope signatures. For *M*. *lucifugus* we selected between 5 and 28 (mean 17) adult females from each of 9 swarming sites (collected over, on average, 7 sampling nights; range 2–25, and 2.3 years; range: 2–3) and between 10 and 14 (mean 12) adult females from 9 summering areas (collected over an average of 2.6 days; range 1–12 and 1.2 years; range 1–3) for analysis. For *M*. *septentrionalis* we selected between 4 and 28 (mean 14) adult females from each of 9 swarming sites (collected over an average of 5.9 days; range 2–20 and 2.6 years; range: 1–4) and between 12 and 15 (mean 14) adult females from 3 summering areas (collected over an average of 10 days; range 9–11 and 2 years; range: 1–3) (Table 1). In addition, fur samples from 34 *M*. *lucifugus* and 31 *M*. *septentrionalis* males captured at one swarming site were analyzed and 7 *M*. *lucifugus* males at one summering area.

**Table 1:** Population level stable isotope metrics derived from *δ* ^13^C and *δ* ^15^N of fur samples of *M*. *lucifugus and M*. *septentrionalis* at summering areas and swarming sites in Nova Scotia.

	*Myotis lucifugus*							*Myotis septentrionalis*						
Summering	N	COSD**	COSY***	NR(‰)	CR(‰)	SEA_c_	TA	N	COSD	COSY	NR(‰)	CR(‰)	SEA_c_	TA
Annapolis Royal	12	1	1	9.12	9.67	19.62	37.52	0						
Antigonish	12	1	1	4.29	5.45	5.02	9.40	0						
Christmas Island	10	1	1	1.21	3.04	1.16	1.69	0						
Dollar Lake	0							15	10	2	2.03	4.20	1.86	4.44
Kejimkujik	14+7*	12	3	4.93	19.22	12.03	28.09	15	11	3	2.14	5.25	3.29	7.73
Millvillage	12	1	1	4.62	22.93	9.71	21.03	0						
Earltown	12	3	1	2.04	5.28	3.56	6.34	12	9	1	1.44	1.02	0.59	1.13
Gore	12	1	1	4.98	2.04	2.31	4.40	0						
Tatamagouche	12	1	1	1.33	1.81	1.14	2.05	0						
West Chezzetcook	12	2	1	5.13	17.76	6.24	11.50	0						
Swarming														
Cave of the Bats	7	3	2	3.23	17.79	22.94	30.60	20	3	2	2.97	2.10	1.58	4.02
Cheverie	20	5	3	4.52	12.65	11.66	26.00	17	5	3	3.58	2.35	1.82	4.86
Donkin	0							4	4	2	0.72	2.35	0.66	0.42
Glenelg	16	2	2	2.90	11.74	6.84	17.69	6	3	2	1.16	1.50	0.68	0.74
Hayes Cave	20	8	2	7.82	25.93	26.09	75.13	19	7	3	4.45	3.13	2.32	7.12
Lake Charlotte	20	6	3	4.53	16.48	9.03	26.26	7	4	3	1.08	2.63	1.63	1.96
Lear Shaft	20	9	2	7.79	30.37	36.53	124.27	19	5	3	2.97	2.44	1.84	4.24
Minasville	5	3	2	4.77	13.90	39.26	32.93	7	2	1	2.10	1.53	1.37	1.79
Rawdon	28+34*	25	3	7.30	30.28	31.62	110.46	28+31*	20	4	3.23	2.23	1.55	5.38
Vault	20	2	2	10.38	21.37	44.48	123.30	0						

* Number of males sampled

** Collected over number of sampling days

*** Collected over number of sampling years

### Stable isotope analysis

Fur samples were analyzed for *δ* ^13^C and *δ* ^15^N at the Stable Isotopes in Nature Laboratory at the University of New Brunswick where they were washed by soaking them in 2:1 (v/v) chloroform:methanol for 10–15 minutes. With clean tweezers samples were stirred and removed from the vial. This method was repeated three times before samples were left to air-dry under a fume hood overnight. Once dried, samples were ground to a fine powder, placed in tin capsules, and weighed to the nearest 0.001 mg (mean: 0.705 mg, SD: 0.308 mg). The minimum carbon amplitude and nitrogen amplitude of our standards were 0.379 V and 0.406 V, respectively, and samples outside of these ranges were not used. Samples were then combusted at 1000°C in ThermoQuest CE Instruments NC2500 Element Analyser (ThermoQuest Italia, Rodano, Italy) and subjected to mass spectrometry with a Thermoquest Finnigan-Mat Delta Plus Continuous Flow Mass Spectrometer (ThermoFinnigan, Bremen, Germany). Duplicate measures were done on 15 randomly selected samples and difference between these measures were on average 0.09‰ (range: 0.01–0.52‰) for *δ* ^13^C and 0.05‰ (range: 0.00–0.10‰) for *δ* ^15^N. Stable isotope measurements are reported as *δ* in parts per thousand (‰) and anchored to the VPBD (δ13C) and AIR (δ15N) scales using calibrated standards [International Atomic Energy Agency: CH6 (-10.4‰), CH7 (-32.2‰), N1 (0.4‰), N2 (20.3‰)]. Isotopic data are reported as *δ*X values (where X represents the heavier isotope ^13^C or ^15^N) as they differ from their standard, and are calculated according to the formula:

$$\delta X=\left[\left(R_{sample}/R_{standard}\right)-1\right]x1000$$

where *R*_sample_ = ^13^C/^12^C or ^15^N/^14^N of the sample, and *R*_standard_ = ^13^C/^12^C of VPDB or ^15^N/^14^N of AIR [63].

### Statistical analyses and metrics

Generalized linear models were used to test if forearm length, sex, or their interaction would explain variation in isotopic ratios (*δ* ^13^C ~ forearm + sex + forearm:sex; *δ* ^15^N ~ forearm + sex + forearm:sex). This was done for both species at Rawdon mine swarming site and for *M*. *lucifugus* at Kejimkujik summering area. Isotopic metrics of *δ* ^13^C and *δ* ^15^N were calculated using the Stable Isotope Analysis in R (SIAR) package [64] and the integrated Stable Isotope Bayesian Ellipses in R (SIBER) package [65] using R version 3.0.2 (The R Foundation for Statistical Computing 2013). Several metrics [66] were used to make inferences about migration dynamics including *δ* ^13^C range (CR) and *δ* ^15^N range (NR), which provides information on the isotopic range of a sampled population. Small sample size corrected standard ellipse area (SEA_c_) represents 40% of the total area (TA), which is the convex hull area encompassing a sampled population in *δ* ^13^C and *δ* ^15^N bi-plot space and is a measure of total isotopic niche space occupied. Overall CR and NR were calculated as the difference between the highest isotopic value and the lowest isotopic value, measured per species within the entire dataset. Separate CR and NR were also calculated per sampled site (Table 1). These data were tested for normality using a Shapiro-Wilk test [67] and further analyzed with either an independent t-test or Mann-Whitney *U* test. The degree of overlap between populations’ SEA_c_ is a function, at least in part, of overlap in prey and environment of origin [65,66]. Overlap of SEA_c_ were calculated in SIAR and spatial distance between sampling sites was calculated in ArcGIS 10.1 (ESRI Inc, Redlands, California, USA), using the point distance tool in spatial analyst. These data were output as matrices and compared using a Mantel test.

To test for interspecific variation in isotopic niche-width between *M*. *lucifugus* and *M*. *septentrionalis*, we calculated the Bayesian standard ellipse area (bootstrapped *n* = 10,000) per species using all sampled individuals. Ellipse areas were statistically compared by calculating the probability that the ellipse of *M*. *lucifugus* is larger than the ellipse of *M*. *septentrionalis*.

Through descriptive discriminant analyses, summering bats were statistically reassigned to summering areas and swarming bats were reassigned to swarming sites to make inferences about degree of isotopic separation between sites. In addition, predictive discriminant analyses [33,34,66] were computed with Systat 12 (Systat Software, Inc.) to statistically reassign swarming bats to summering areas within the dataset to which they had the greatest affinity using *δ* ^13^C and *δ* ^15^N profiles. This method was used to make inferences about whether bats at swarming sites originate from one or multiple summering areas.

## Results

There was no evidence of a significant effect of forearm length, sex, or interaction of the two on *δ* ^13^C and *δ* ^15^N of either *M*. *lucifugus* and *M*. *septentrionalis* (all Ps > 0.05). Regardless, because males were only sampled at one swarming site and one summering area, all subsequent analyses incorporated only females.

There was no correlation between SEA_c_ overlap and distance between sites for *M*. *lucifugus* (Mantel test: 999 replicates, *R* ^*2*^: -0.067, *P* = 0.649) or *M*. *septentrionalis* (Mantel test: 999 replicates, *R* ^*2*^: 0.336, *P* = 0.509).

**Fig 2:**
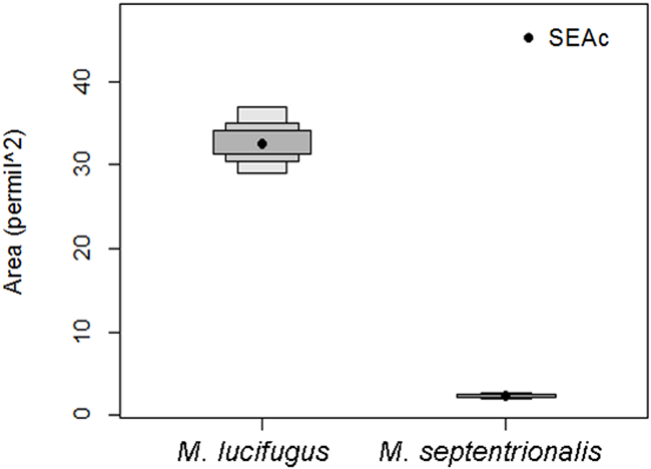
Estimated posterior distributions of *M*. *lucifugus* (left) and *M*. *septentrionalis* (right) with 50%, 75% and 95% credible intervals.

**Fig 3:**
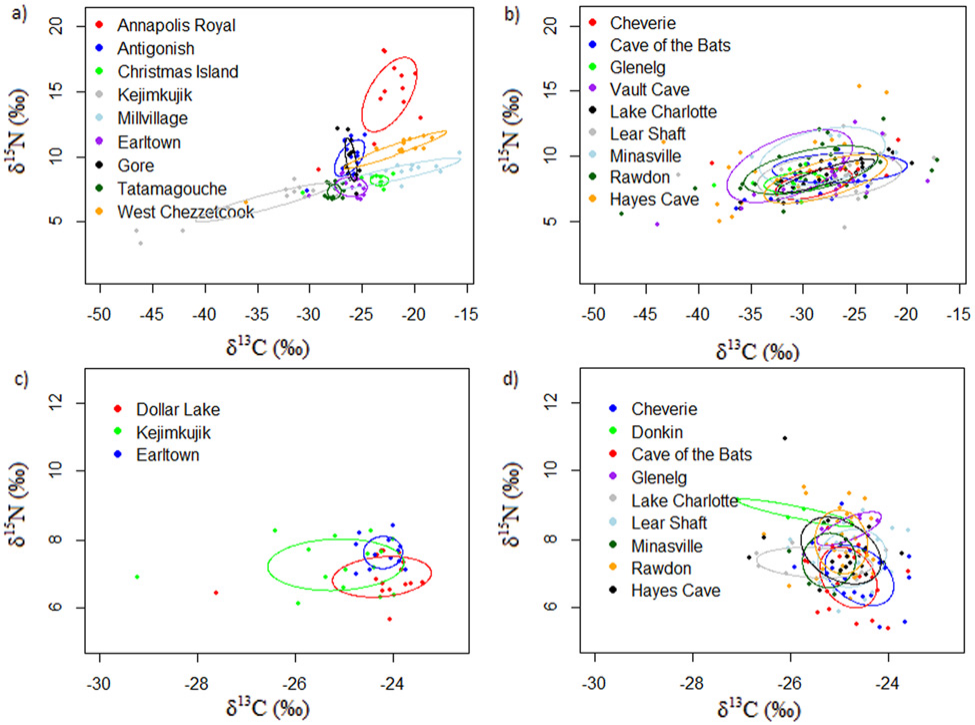
Scatter plots of *δ* ^13^C ‰ and *δ* ^15^N ‰ values of *M*. *lucifugus* (a: summering) (b: swarming) and *M*. *septentrionalis* (c: summering) (d: swarming) fur samples in Nova Scotia where ovals enclose the small sample size standard ellipse area (SEA_c_; 40%).

Bayesian statistics indicated a 100% probability that the SEA_c_ of all sampled *M*. *lucifugus* is larger than the SEA_c_ of all sampled *M*. *septentrionalis* (Fig 2). For *M*. *lucifugus* the overall CR was 32.14‰ (30.70‰ for swarming sites and 30.79‰ for summering areas) but there was considerable variation among sites (Table 1). For example, the CR at summering areas ranged from 1.81‰ (Tatamagouche) to 22.93‰ (Mill Village). Overall NR was 14.82‰ (10.84‰ for swarming sites and 14.82‰ for summering areas) and there was less variability within summering areas ranging from 1.21‰ (Christmas Island) to 9.12‰ (Annapolis Royal). For *M*. *septentrionalis* the overall CR was 5.82‰ (3.51‰ for swarming sites and 5.82‰ for summering areas) and variability in CR within summering areas ranged from 1.02‰ (Earltown) to 5.82‰ (Kejimkujik). The overall NR was 5.57‰ for *M*. *septentrionalis* (5.57‰ for swarming sites and 2.77‰ for summering areas) and there was little variation among the summering areas (Table 1; Fig 3).

For *M*. *lucifugus*, the distribution of SEA_c_ at sample locations was not normal (Shapiro-Wilk test: *P* = 0.026, *df* = 18) and were significantly larger at swarming sites than at summering areas (Mann-Whitney: *U* = 8.000, *P* = 0.003, *Z* = -2.870; Fig 3 and S1 Table). For *M*. *septentrionalis* SEA_c_ were normally distributed (Shapiro-Wilk test: *P* = 0.229, *df* = 12) and showed no significant difference between summering areas and swarming sites (Independent t-test: *t* = -0.814, *P* = 0.435, *df* = 10; Fig 3 and S2 Table).

Descriptive discriminant analyses correctly reassigned, on average, 62.2% of *M*. *lucifugus* summering area samples to their origin (Table 2) and only 18.9% of the swarming samples to their capture site (Table 3). Bats from the summering area at West Chezzetcook were correctly reassigned most often (92% correct) and bats from Gore were most often misassigned (8% correct). For *M*. *septentrionalis*, on average, 58.3% of summering bats were correctly reassigned to their origin (Table 4) but only 29.6% of swarming bats were correctly reassigned to their capture site (Table 5). Bats from the summering area at Earltown were most often correctly reclassified (75% correct) and bats from Kejimkujik were most frequently misclassified (40% correct).

**Table 2:** Classification matrix of *M*. *lucifugus* correctly assigned to their known summering area based on *δ* ^13^C and *δ* ^15^N isotopic signatures.

To →	N	Annapolis Royal	Antigonish	Christmas Island	Earltown	Kejimkujik	Mill village	Gore	Tatama gouche	West Chezzetcook	% Correct
From ↓											
Annapolis Royal	12	9	2	0	0	0	0	0	0	1	75
Antigonish	12	0	7	0	2	0	0	2	0	1	58
Christmas Island	10	0	0	6	1	0	2	0	1	0	60
Earltown	12	0	0	4	5	0	0	2	1	0	42
Kejimkujik	14	0	0	0	0	11	0	0	3	0	79
Millvillage	12	0	0	0	0	10	0	0	4	0	71
Gore	12	1	5	0	5	0	0	1	0	0	8
Tatamagouche	12	0	0	0	3	0	0	0	9	0	75
West Chezzetcook	12	0	0	0	0	1	0	0	0	11	92

**Table 3:** Classification matrix of *M*. *lucifugus* correctly assigned to their swarming capture sites based on *δ* ^13^C and *δ* ^15^N isotopic signatures.

To →	N	Cave of the Bats	Cheverie	Glenelg	Hayes Cave	Lake Charlotte	Lear Shaft	Minasville	Rawdon	Vault Cave	% Correct
From ↓											
Cave of the Bats	7	2	0	0	1	0	2	1	0	1	29
Cheverie	20	2	5	5	4	0	1	1	2	0	25
Glenelg	16	1	5	5	0	0	0	0	1	4	31
Hayes Cave	20	3	4	5	1	1	3	3	0	0	5
Lake Charlotte	20	6	2	4	3	1	1	1	2	0	5
Lear Shaft	20	3	3	0	6	0	3	2	0	3	15
Minasville	5	1	1	0	0	0	0	2	0	1	40
Rawdon	28	5	1	6	2	0	0	9	0	5	0
Vault Cave	20	2	0	6	1	1	0	5	1	4	20

**Table 4:** Classification matrix of *M*. *septentrionalis* correctly assigned to their known summering area based on *δ* ^13^C and *δ* ^15^N isotopic signatures.

To →	N	Dollar Lake	Earltown	Kejimkujik	% Correct
From ↓					
Dollar Lake	15	9	5	1	60
Earltown	12	3	9	0	75
Kejimkujik	15	4	5	6	40

**Table 5:** Classification matrix of *M*. *septentrionalis* correctly assigned to their swarming capture sites based on *δ* ^13^C and *δ* ^15^N isotopic signatures.

To →	N	Cave of the bats	Cheverie	Donkin	Glenelg	Hayes Cave	Lake Charlotte	Lear Shaft	Minasville	Rawdon	% Correct
From ↓											
Cave of the Bats	20	4	6	0	0	2	3	2	1	2	20
Cheverie	17	5	4	0	1	0	2	3	2	0	24
Donkin	4	0	0	3	1	0	0	0	0	0	75
Glenelg	6	0	0	0	4	0	1	0	0	1	67
Hayes Cave	19	2	4	2	2	0	2	5	1	1	0
Lake Charlotte	7	0	2	1	0	0	3	1	0	0	43
Lear Shaft	19	2	3	1	6	0	3	3	0	1	16
Minasville	7	2	0	0	1	0	2	1	0	1	4
Rawdon	28	2	3	3	9	3	3	4	0	1	17

Predictive discriminant analysis suggested that *M*. *lucifugus* captured at swarming sites had originated from many summering areas as, within our analyses, bats from each swarming site were reassigned, on average, to 6.2 (out of 9) of our sampled summering areas. Based on these reassignments, Minasville ice cave and Glenelg mine swarming congregations were the least diverse as individuals from these sites were reassigned to the fewest summering areas within our dataset (3 and 4 respectively; Table 6). For *M*. *septentrionalis*, on average, samples from a swarming site were reassigned to 2.8 (out of 3 that were sampled) summering areas. Donkin mine and Glenelg mine swarming congregations were reassigned to Kejimkujik and Earltown summering areas, whereas bats from all other swarming sites were reassigned to 3 sampled summering areas (Table 7).

**Table 6:** Number of *M*. *lucifugus* from swarming sites assigned to summering areas with predictive discriminant analysis.

To →	N	Annapolis Royal	Anti gonish	Christmas Island	Keji mkujik	Mill village	Earltown	Gore	Tatama gouche	West Chezzet cook	# sites assigned to
From ↓											
Cheverie	20		3	4		6		2	5		5
Cave of the Bats	7		1	2			1	1	1	1	6
Glenelg	16		2			8		2	4		4
Vault Cave	20	3	6		1	6		4			5
Lake Charlotte	20		2	2		6	1	4	3	2	7
Lear Shaft	20	1	1	4	4	3	1	1	3	2	9
Minasville	5		3			1				1	3
Rawdon	28	2	9	1	1	10	2	2	0	1	8
Hayes Cave	20	1	1	1	4	5	1	3	2	2	9

**Table 7:** Number of *M*. *septentrionalis* from swarming sites assigned to summering areas with predictive discriminant analysis.

To →	N	Dollar Lake	Keji mkujik	Earltown	# sites assigned to
From ↓					
Cheverie	17	9	4	4	3
Donkin	4		2	2	2
Cave of the Bats	20	11	4	5	3
Glenelg	6		5	1	2
Lake Charlotte	7	2	1	4	3
Lear Shaft	19	5	8	6	3
Minasville	7	1	3	3	3
Rawdon	28	4	15	9	3
Hayes Cave	19	4	8	7	3

## Discussion

In this study we investigated whether *δ* ^13^C and *δ* ^15^N from fur could be used to make inferences about regional migration of two bat species by applying predictive discriminant analyses. Considerable interspecific variation in isotopic niche area was detected in *M*. *lucifugus* and *M*. *septentrionalis*. *Myotis lucifugus* has a larger isotopic niche than *M*. *septentrionalis*, supporting the contention that they are a more generalist forager [47,48,57].

No effects of sex or forearm length on stable isotope ratios were detected within our dataset. However, males were only sampled at a limited number of sites and more thorough sampling of both males and females needs to be done, ideally at summering areas, to make more meaningful inference about the potential effect of sex on niche dynamics using stable isotopes and to assess if data from males and females can be pooled for additional analyses. No effect of distance between sites on stable isotope ratios was detected.

The SEA_c_ of Annapolis Royal was most different from other summering areas due to high *δ* ^15^N values. Annapolis Royal, in the Annapolis valley, is an area with intensive agriculture [68]. Fertilizer is high in nitrogen and run-off from agriculture leaches into the surrounding environment and thus *δ* ^15^N enters the food chain [69–72]. The sampled swarming site nearest Annapolis Royal is Vault Cave and interestingly this site, located in a diverse environment of coastal forest and agriculture [68], showed a higher NR than other swarming sites. Despite this observation more individuals from Vault Cave were reassigned to summering areas other than Annapolis Royal (Antigonish, Mill Village and Gore), likely because, despite its broad NR profile, it did not completely overlap the range of Annapolis Royal.

For *M*. *lucifugus* SEA_c_ was significantly larger at swarming sites than at individual summering areas and there was more variation in profiles among summering areas than among swarming sites. These results support the contention that congregations of bats at swarming sites consist of bats that have originated from multiple summering areas. Molecular and mark-recapture methods have provided similar evidence for mixing at swarming sites in other bat species [73,74,75]. No such differences were detected for *M*. *septentrionalis* in this study but because of the low number of summering areas, and very low intraspecific variation, inference power was limited.

Samples from summering areas for both *M*. *lucifugus* and *M*. *septentrionalis* were correctly reassigned at a higher frequency than individuals sampled at swarming sites, although this effect was weak for *M*. *septentrionalis*. This suggests that the isotopic profiles of at least *M*. *lucifugus* at summering areas were more distinct than those at swarming sites, likely because swarming sites consist of individuals originating from multiple summering areas. Alternatively, temporal variation in isotopic ratios could explain why swarming congregations had wider isotopic ranges. In this study, samples from swarming sites were collected, on average, over longer times than samples from summering areas. However, the isotopic ranges do not appear to be consistent with ranges of sample collection and thus temporal variation is not believed to be large enough to be a major contributor to these results. Furthermore, predictive discriminant analysis always assigns all cases to one of the provided groups, even if the fit to these groups is poor. Therefore, in this study discriminant analysis gives an indication of the diversity of summering colonies contributing to swarming congregations, rather than which summering colonies contribute. Regardless, these data suggest that swarming sites for bats have a catchment area of summering areas that include multiple colonies.

Our results indicate that individuals sampled at swarming sites originated from many different summering areas, at least for *M*. *lucifugus*. Females of *M*. *lucifugus* and *M*. *septentrionalis* are known to show high fidelity to summering areas which could result in isolation of populations and thus low genetic variation [47,74]. However, the mixing that occurs at swarming sites promotes gene flow and therefore, increased genetic diversity [76]. Bat movement dynamics appear tortuous due to the mixing of summering individuals at swarming sites. Bats may travel tens to hundreds of kilometers [37–39] to reach swarming sites, even if other swarming sites are closer. Although there is still much to learn about fur moulting in bats, our data indicate that *δ* ^13^C and *δ* ^15^N analysis of the fur of even regionally migrating bat species can be used to make general inferences about the origin diversity of at least some bat species at swarming sites during local fall migration. Increasing the number of isotopes in an analysis (e.g. *δ* ^34^S) may permit even more detailed inference about their migration.

## Supporting Information

S1 Table Matrix of SEA_c_ overlap between sites (area of overlap/area of larger ellipse) and spatial distances (in kilometers: lower half) comparing all *M*. *lucifugus* summering areas and swarming sites in Nova Scotia. (DOCX)

S2 Table Matrix of SEA_c_ overlap between sites (area of overlap/area of larger ellipse) and spatial distances (in kilometers: lower half) comparing all *M*. *septentrionalis* summering areas and swarming sites in Nova Scotia. (DOCX)
