# Supplementary material for: Carbon (δ13C) and Nitrogen (δ15N) Stable Isotope Signatures in Bat Fur Indicate Swarming Sites Have Catchment Areas for Bats from Different Summering Areas
Source: PLoS One. 2015 Apr 29;10(4):e0125755. doi: 10.1371/journal.pone.0125755 (PMC4414594; doi:10.1371/journal.pone.0125755)
Supplement: S1 Table — (DOCX) [file pone.0125755.s001.docx]

#### S1 Table. Matrix of SEA_c_ overlap between sites (area of overlap/area of larger ellipse) and spatial distances (in kilometers: lower half) comparing all *M. lucifugus* summering areas and swarming sites in Nova Scotia.

|  |  | Summering | | | | | | | | |  | Swarming | | | | | | | | |
| --- | --- | --- | --- | --- | --- | --- | --- | --- | --- | --- | --- | --- | --- | --- | --- | --- | --- | --- | --- | --- |
| Summering | | Anna polis Royal | Antigonish | Christmas Island | Kejim kujik | Mill village | Earl town | Gore | Tatamagouche | West Chezzetcook |  | Chev erie | Cave of the Bats | Glenelg | Vault Cave | Lake Char lotte | Lear Shaft | Minas ville | Raw don | Hayes Cave |
|  | Annapolis Royal |  | 0.000 | 0.000 | 0.000 | 0.000 | 0.000 | 0.000 | 0.000 | 0.000 |  | 0.000 | 0.000 | 0.000 | 0.000 | 0.000 | 0.000 | 0.004 | 0.000 | 0.000 |
|  | Antigonish | 283 |  | 0.000 | 0.000 | 0.000 | 0.000 | 0.321 | 0.000 | 0.094 |  | 0.056 | 0.000 | 0.000 | 0.080 | 0.149 | 0.088 | 0.128 | 0.170 | 0.101 |
|  | Christmas Island | 395 | 112 |  | 0.000 | 0.098 | 0.000 | 0.000 | 0.000 | 0.000 |  | 0.000 | 0.027 | 0.000 | 0.000 | 0.000 | 0.032 | 0.000 | 0.000 | 0.019 |
|  | Kejimkujik | 49 | 278 | 389 |  | 0.061 | 0.068 | 0.000 | 0.053 | 0.000 |  | 0.422 | 0.014 | 0.156 | 0.081 | 0.143 | 0.138 | 0.006 | 0.106 | 0.240 |
|  | Millvillage | 94 | 254 | 365 | 47 |  | 0.170 | 0.009 | 0.017 | 0.000 |  | 0.097 | 0.188 | 0.000 | 0.000 | 0.003 | 0.162 | 0.000 | 0.068 | 0.136 |
|  | Earltown | 199 | 92 | 197 | 204 | 194 |  | 0.028 | 0.069 | 0.000 |  | 0.133 | 0.042 | 0.000 | 0.000 | 0.038 | 0.097 | 0.006 | 0.063 | 0.107 |
|  | Gore | 145 | 137 | 247 | 144 | 131 | 62 |  | 0.000 | 0.057 |  | 0.038 | 0.069 | 0.000 | 0.032 | 0.091 | 0.043 | 0.056 | 0.080 | 0.052 |
|  | Tatamagouche | 195 | 104 | 205 | 204 | 197 | 15 | 67 |  | 0.000 |  | 0.067 | 0.000 | 0.000 | 0.000 | 0.002 | 0.031 | 0.000 | 0.017 | 0.041 |
|  | West Chezzetcook | 177 | 129 | 239 | 158 | 127 | 95 | 57 | 106 |  |  | 0.000 | 0.089 | 0.000 | 0.004 | 0.093 | 0.053 | 0.070 | 0.066 | 0.073 |
| Swarming | |  |  |  |  |  |  |  |  |  |  |  |  |  |  |  |  |  |  |  |
|  | Cheverie | 113 | 170 | 279 | 120 | 118 | 84 | 36 | 83 | 86 |  |  | 0.216 | 0.420 | 0.147 | 0.553 | 0.318 | 0.137 | 0.364 | 0.447 |
|  | Cave of the Bats | 165 | 122 | 234 | 155 | 135 | 65 | 24 | 75 | 35 |  | 59 |  | 0.100 | 0.255 | 0.286 | 0.585 | 0.414 | 0.725 | 0.569 |
|  | Glenelg | 272 | 28 | 132 | 263 | 234 | 95 | 125 | 110 | 108 |  | 161 | 107 |  | 0.147 | 0.423 | 0.187 | 0.099 | 0.228 | 0.235 |
|  | Vault Cave | 47 | 239 | 349 | 75 | 101 | 152 | 104 | 147 | 143 |  | 69 | 125 | 230 |  | 0.116 | 0.388 | 0.625 | 0.361 | 0.262 |
|  | Lake Charlotte | 199 | 104 | 212 | 183 | 152 | 81 | 63 | 95 | 26 |  | 98 | 37 | 82 | 162 |  | 0.247 | 0.183 | 0.363 | 0.346 |
|  | Lear Shaft | 166 | 124 | 229 | 174 | 168 | 32 | 39 | 29 | 91 |  | 54 | 56 | 122 | 121 | 86 |  | 0.451 | 0.561 | 0.669 |
|  | Minasville | 145 | 139 | 249 | 149 | 141 | 55 | 17 | 55 | 76 |  | 30 | 42 | 133 | 101 | 78 | 26 |  | 0.493 | 0.316 |
|  | Rawdon | 133 | 148 | 260 | 132 | 119 | 73 | 12 | 76 | 60 |  | 28 | 32 | 137 | 94 | 69 | 50 | 23 |  | 0.606 |
|  | Hayes Cave | 165 | 119 | 228 | 164 | 150 | 41 | 19 | 47 | 63 |  | 51 | 29 | 111 | 100 | 59 | 26 | 22 | 33 |  |
