# Supplementary material for: Carbon (δ13C) and Nitrogen (δ15N) Stable Isotope Signatures in Bat Fur Indicate Swarming Sites Have Catchment Areas for Bats from Different Summering Areas
Source: PLoS One. 2015 Apr 29;10(4):e0125755. doi: 10.1371/journal.pone.0125755 (PMC4414594; doi:10.1371/journal.pone.0125755)
Supplement: S2 Table — (DOCX) [file pone.0125755.s002.docx]

#### S2 Table. Matrix of SEA_c_ overlap between sites (area of overlap/area of larger ellipse) and spatial distances (in kilometers: lower half) comparing all *M. septentrionalis* summering areas and swarming sites in Nova Scotia.

|  |  | Summering | | |  | | Swarming | | | | | | | | |  |
| --- | --- | --- | --- | --- | --- | --- | --- | --- | --- | --- | --- | --- | --- | --- | --- | --- |
| Summering | | Dollar Lake | Keji mkujik | Earl town | |  | Chev erie | Don kin | Cave of the Bats | Glen elg | Lake Char lotte | Lear Shaft | Minas ville | Raw don | Hayes Cave |  |
|  | Dollar Lake |  | 0.298 | 0.101 | |  | 0.642 | 0.000 | 0.456 | 0.000 | 0.166 | 0.381 | 0.139 | 0.087 | 0.255 |  |
|  | Kejimkujik | 161 |  | 0.131 | |  | 0.383 | 0.000 | 0.340 | 0.031 | 0.474 | 0.482 | 0.391 | 0.103 | 0.505 |  |
|  | Earltown | 71 | 204 |  | |  | 0.100 | 0.000 | 0.020 | 0.000 | 0.026 | 0.218 | 0.000 | 0.126 | 0.121 |  |
| Swarming | |  |  |  | |  |  |  |  |  |  |  |  |  |  |  |
|  | Cheverie | 71 | 120 | 84 | |  |  | 0.000 | 0.700 | 0.000 | 0.328 | 0.604 | 0.315 | 0.307 | 0.439 |  |
|  | Donkin | 303 | 465 | 273 | |  | 355 |  | 0.000 | 0.072 | 0.000 | 0.000 | 0.000 | 0.058 | 0.051 |  |
|  | Cave of the Bats | 12 | 155 | 65 | |  | 59 | 309 |  | 0.000 | 0.409 | 0.535 | 0.436 | 0.287 | 0.385 |  |
|  | Glenelg | 100 | 263 | 95 | |  | 161 | 203 | 107 |  | 0.000 | 0.112 | 0.123 | 0.357 | 0.182 |  |
|  | Lake Charlotte | 27 | 183 | 81 | |  | 98 | 286 | 37 | 82 |  | 0.392 | 0.517 | 0.281 | 0.368 |  |
|  | Lear Shaft | 65 | 174 | 32 | |  | 54 | 305 | 56 | 122 | 86 |  | 0.461 | 0.602 | 0.686 |  |
|  | Minasville | 52 | 149 | 55 | |  | 30 | 324 | 42 | 133 | 78 | 26 |  | 0.403 | 0.461 |  |
|  | Rawdon | 43 | 132 | 73 | |  | 28 | 334 | 32 | 137 | 69 | 50 | 23 |  | 0.578 |  |
|  | Hayes Cave | 38 | 164 | 41 | |  | 51 | 303 | 29 | 111 | 59 | 26 | 22 | 33 |  |  |
